# Association between small intestine bacterial overgrowth and psychiatric disorders

**DOI:** 10.3389/fendo.2024.1438066

**Published:** 2024-10-21

**Authors:** Bartosz Bogielski, Katarzyna Michalczyk, Piotr Głodek, Bartosz Tempka, Wojciech Gębski, Dominika Stygar

**Affiliations:** Department of Physiology, Faculty of Medical Sciences in Zabrze, Medical University of Silesia, Zabrze, Poland

**Keywords:** gut-brain axis, psychiatric disorder, SIBO, small intestine bacterial overgrowth, thyroid disorder, tryptophan pathway

## Abstract

Small intestinal bacterial overgrowth (SIBO) is a gastrointestinal condition characterized by abnormal colonization of bacteria in the small intestine, leading to overgrowth and alteration, which is linked to gastrointestinal issues, potentially affecting neurological and mental health. Despite existing research, we still do not understand how SIBO affects tryptophan metabolism and psychiatric diseases. We investigated the literature for connections between SIBO, tryptophan metabolism disruptions, and psychiatric disorders like autism, schizophrenia, Alzheimer’s, and Parkinson’s diseases. We also explored the interaction between thyroid disorders and their influence on SIBO and psychiatric illnesses. PubMed and Google Scholar databases were searched using keywords and phrases, individual and in combinations, like “SIBO,” “gut microbiota,” “neurologic disorders,” “mental disorders,” “tryptophan,” “dopamine,” and “thyroid disease.” We focused on original research and review papers that presented empirical studies conducted on animal models and human subjects published in English between February 1992 to February 2023. The initial 2 634 534 records were preliminary screened based on title and abstract and then subjected to full-text review to exclude publications with insufficient data on SIBO, lack of a psychiatric disorder component, or methodological limitations compromising the integrity of the findings. The analysis highlights the significance of the association between psychiatric disorders and SIBO, emphasizing the role of gut-microbial diversity in mental health. We advocate for more detailed studies, including longitudinal research, to clarify the causal relationships between SIBO, gut dysbiosis, and psychiatric disorders and for an integrated approach while treating complex psychiatric conditions.

## 1 Introduction

Small intestinal bacterial overgrowth (SIBO) is a medical condition characterized by the excessive accumulation and alteration of bacterial populations in the small intestine (1). This imbalance, known as dysbiosis, disrupts the equilibrium between beneficial commensal bacteria and harmful pathogenic bacteria within the intestinal ecosystem (2). This condition can lead to gastrointestinal and systemic issues, including bloating, diarrhea, malnutrition, and abdominal pain (3–5). It can also impact functional gastrointestinal disorders and potentially alter the presentation of chronic diseases like heart failure and diabetes (6).

Recent studies mention the potential impact of SIBO on neurological and mental health and highlight a bidirectional relationship between the gut microbiome and brain functionality (7–14). Additionally, studies have indicated a significant interaction between psychiatric disorders such as generalized anxiety disorder, panic disorder, major depressive disorder, bipolar disorder, schizophrenia, and irritable bowel syndrome (IBS) (15). The co-occurrence of IBS with common mental disorders, particularly anxiety, and depression, is well documented, suggesting shared biological and psychosocial disease mechanisms, many of which contribute to a dysregulated gut-brain axis (4).

Furthermore, reduced gut-microbial diversity, or gut dysbiosis, has been associated with various mental disorders, indicating a potential underlying factor contributing to these conditions (16). A cross-sectional study has also explored the high psychiatric co-morbidity in patients with functional gastrointestinal disorders, emphasizing the need for a better understanding of these relationships (17).

The genetic aspects of these associations have been studied as well (18). For instance, IBS, a chronic disorder of gut-brain interaction, often co-occurs with mental conditions like depression and anxiety (19). Although heritable factors play a role, the exact genetic underpinnings explaining the high rates of comorbidity remain unclear (5).

Overall, these studies underscore the complex interplay between gut health, particularly SIBO and gut dysbiosis, and various psychiatric conditions, highlighting the importance of considering these factors in the development of screening and treatment strategies for psychiatric disorders.

The relationship between SIBO and various psychiatric disorders is intricate (7). The presented review focuses on the potential links between SIBO, disruptions in tryptophan metabolism, and the onset or exacerbation of specific psychiatric conditions, including autism, schizophrenia, Alzheimer’s, and Parkinson’s diseases (20, 21). Furthermore, the study explores the correlation between thyroid disorders and psychiatric disorders (22–24). Thyroid hormones, triiodothyronine and thyroxine, play an integral role in neuronal growth, metabolism, and neurotransmitter production, and are linked to some neurological and mental illnesses (25–29). Therefore, hyperthyroidism and hypothyroidism could have a potential influence on SIBO and psychiatric illnesses. Many studies link SIBO and psychiatric disorders, but only a few studies point directly to the cause of these disorders in patients with SIBO (26, 30–32). On the other hand, SIBO is known to promote the onset of thyroid diseases (33). Based on the symptomatology of thyroid diseases and related endocrine disorders, it is probable that SIBO might indirectly, through thyroid pathology, affect the mental disorders of patients, but specific research would be required.

Despite the increasing interest in researching the connection between small intestinal bacterial overgrowth and psychiatric disorders and increasing evidence supporting the critical role of the gut-brain axis in these conditions the significant gaps in the knowledge still exist. Firstly, the precise mechanisms underlying the interaction between SIBO and the development or exacerbation of psychiatric conditions are not fully understood (34, 35). Particularly, those associated with the tryptophan pathway, such as autism, schizophrenia, Alzheimer’s, and Parkinson’s diseases. There is a need for more detailed studies that specifically investigate the influence of SIBO on the metabolism of tryptophan and its downstream effects on neurotransmitter production and brain function (14, 36). Additionally, while the correlation between thyroid disorders and psychiatric illnesses is recognized, the role of SIBO in this relationship is less clear (37–39). There is a lack of research exploring how SIBO might affect thyroid function and in turn, how this interaction could influence psychiatric disease processes. This gap is particularly critical considering the potential implications for treatment strategies that target not just the psychiatric symptoms, but also the underlying gut and thyroid dysfunctions. Moreover, there is a scarcity of longitudinal studies that could provide insights into the temporal relationships between SIBO, gut dysbiosis, and the onset or progression of psychiatric disorders. Such studies are essential to determine whether SIBO is a cause, consequence, or coincidental occurrence relative to these mental health conditions.

The presented review aims to enhance the understanding of the complex interactions between gut microbiota and neurological pathways, especially those related to the tryptophan pathway and endocrine imbalances. Thereby, it offers new perspectives on the etiology by examining the potential interactions between SIBO and thyroid disorders, and it indicates potential therapeutic approaches for these psychiatric conditions.

## 2 Methodology for data gathering

The content of this mini-review was gathered by searching the PubMed and Google Scholar databases to collate and analyze the extent of literature on the association between small intestine bacterial overgrowth (SIBO) and various psychiatric disorders. A comprehensive search strategy was employed using a combination of keywords and phrases, including but not limited to “SIBO,” “gut microbiota,” “neurologic disorders,” “mental disorders,” “tryptophan,” “dopamine,” and “thyroid disease.” These terms were used individually and in various combinations to ensure a broad yet relevant coverage of the subject matter.

The search was specifically tailored to include original research articles and review papers that presented empirical studies conducted on both animal models and human subjects. The time frame for publication was set from February 1992 to February 2023, allowing for a broad historical perspective and the inclusion of the most recent findings in the field. To maintain consistency and clarity in our analysis, we limited our search to articles published in English, excluding non-English publications to avoid potential discrepancies arising from translation inaccuracies.

The initial database search yielded: 2 634 534 records from PubMed and Google Scholar. Each article was subjected to a preliminary screening based on title and abstract, focusing on relevance to the study’s objectives and adherence to the inclusion criteria. This screening process, led by two researchers, excluded irrelevant articles and articles not focusing on the direct association between SIBO and psychiatric disorders.

Subsequently, the remaining articles underwent a rigorous full-text review, during which articles were further excluded. The reasons for exclusion at this stage varied, including insufficient data on SIBO, lack of a psychiatric disorder component in the study, or methodological limitations that could compromise the integrity of the findings.

Ultimately, articles were selected for detailed analysis and inclusion in this systematic review. These articles were critically appraised and synthesized to elucidate the complex relationship between SIBO and psychiatric disorders, providing a comprehensive overview of the current state of research in this intriguing area of study.

## 3 Metabolic pathways of tryptophan in patients with SIBO: implications for neurodegenerative and psychiatric disorders

Neurologic and mental disorders include an array of debilitating conditions, i.e., Parkinson’s disease (PD), schizophrenia, major depressive disorder, and anxiety disorders (40, 41). Each one of these diseases exhibits neurochemically and neurophysiologically dysregulated pathways, which contribute to its pathogenesis. Recent years have witnessed increased research into the role neurotransmitters play in neurologic and psychotic functioning (12).

**Figure 1:**
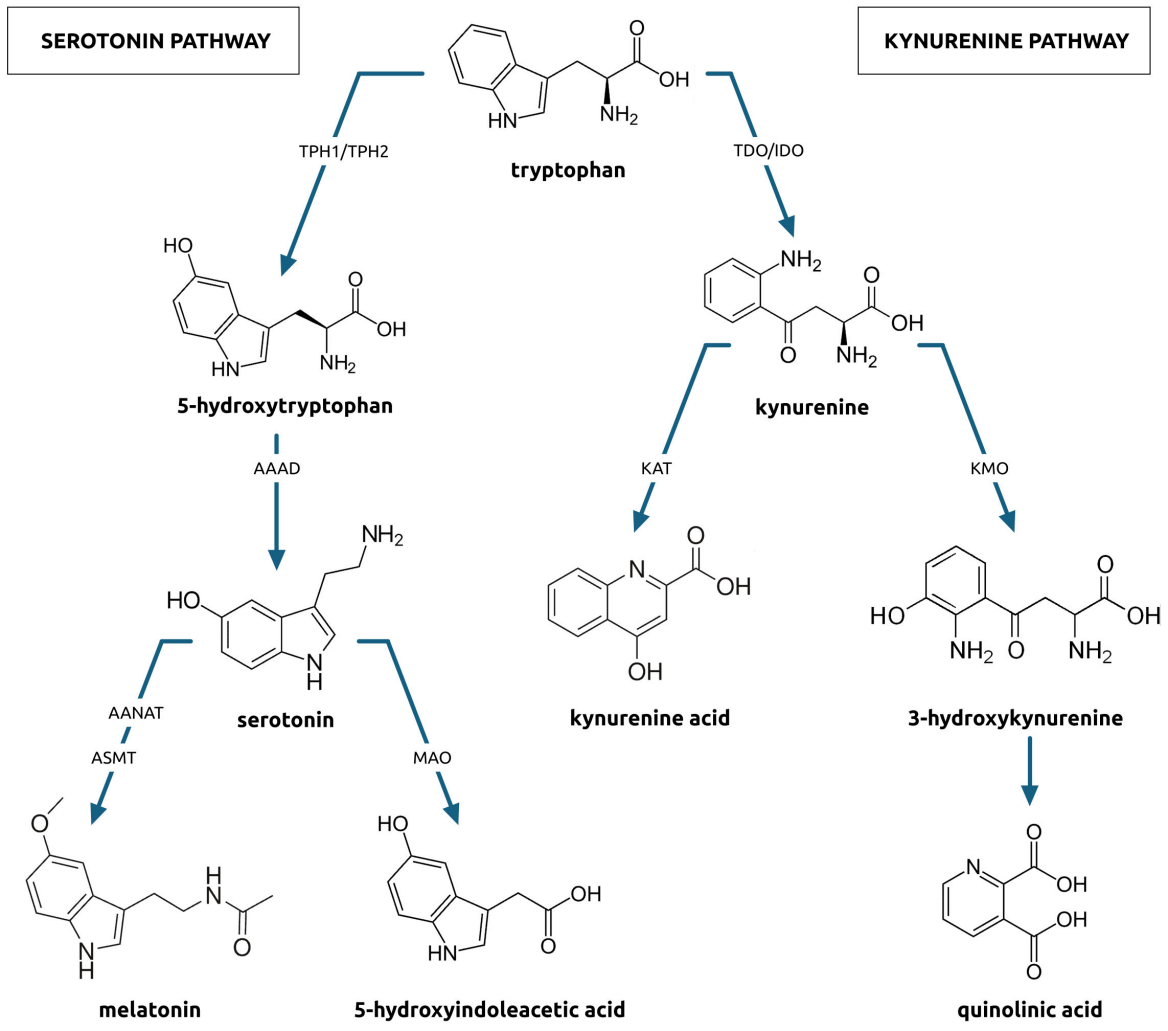
Serotonin and kynurenine pathways of tryptophan metabolism.

Tryptophan (TRP) is metabolized in three pathways. First is the serotonin pathway (**Figure 1**) in which tryptophan is transformed to 5-hydroxytryptophan by tryptophan hydroxylase (TPH). This enzyme exists in two isoforms, TPH1, found in gastroendocrine cells, and, TPH2, found in the central and enteric nervous systems. The 5-hydroxytryptophan is decarboxylated to serotonin (5-HT) by aromatic acid decarboxylase. Further down the pathway, serotonin is transformed two-way (42, 43). The first way comprises a sequence of changes to melatonin, which is an indolamine synchronizing circadian rhythms and regulating sleep initiation. The second way comprises a monoamine oxidase activity and a two-step transformation of serotonin to 5-hydroxyindoleacetic acid (5-HIAA) excreted in the urine. Serotonin is crucial for the proper function of the central nervous system (44). Changes in its metabolism can lead to neurodegenerative or psychiatric diseases (45, 46).

Another pathway is the kynurenine pathway (**Figure 1**) in which tryptophan is transformed into kynurenine (KYN). Kynurenine metabolism affects immune activation, inflammation, and neuroactive molecules production, which can contribute to neurologic or psychiatric conditions development (8). Approximately 90% of the KYN pathway takes part in the liver and is catalyzed by tryptophan 2,3-dioxygenase whose expression can be induced by glucocorticoids. The remaining portion of kynurenine metabolism takes place in the brain, liver, and GI tract, where is catalyzed by indoleamine 2,3-dioxygenase (IDO), whose expression can be induced by cytokines, especially those released during Th1-type immune responses, radicals, and interferons (47). In subsequent stages, kynurenine can be metabolized to kynurenic acid (KA) or quinolinic acid (QA), with 3-hydroxykynurenine (3HK) as an intermediate product. Kynurenic acid acts on multiple receptors in the central nervous system. It is believed to have neuroprotective properties, as it acts as the NMDA receptor antagonist (48). As an agonist, kynurenic acid also shows neuroprotective effects. It can bind with NR1 and NR2 sites of the NMDA receptor, showing affinity to the aryl hydrocarbon receptor and G protein-coupled receptor (16). On the other hand, quinolinic acid, the other product of the KYN pathway, acts as an NMDA receptor agonist that presents neurotoxic effects and enhances free radical production (49). Changes in the activity of this pathway also can lead to neurodegenerative or psychiatric disorders (50).

Recent papers by Chojnacki et al. (7, 8, 51), who evaluated selected metabolites of the tryptophan KYN pathway in the urine of depressive patients with SIBO give grounds to look for links between SIBO and different neurodegenerative or psychiatric disorders co-occurrence (52, 53). It has been shown that patients with SIBO have elevated levels of kynurenine and QA but decreased levels of tryptophan and kynurenic acid in the urine (8), which might have indicated an increased tryptophan use in a neurotoxic pathway leading to quinolinic acid production. Chojnacki et al. (7, 8, 51) found that in patients with SIBO, compared to control patients, the KYN/QA ratio is decreased but the KYN/TRP ratio is elevated, which may indicate an increase in tryptophan degradation and imbalance between neurotoxic and neuroprotective branches of KYN pathways. Moreover, rifaximin treatment in patients with SIBO lowered the KYN/TRP ratio closer to the control group ratio (7). Also, patients with SIBO presented elevated levels of TPH1, 5-HT, and 5-HIAA in the urine (51). The elevation was higher in patients with a diarrheal form of the disease (D-SIBO) than in patients with a constipation form (C-SIBO), however, it was always higher than in healthy patients (51). Moreover, the urine level of 5-HIAA correlated with the lactulose hydrogen breath test (LHBT) result in both groups. Rifaximin treatment decreased the urinary 5-HIAA concentration and the LHBT results, which indicated a direct therapeutic impact of microbiome modulation on the serotonin metabolic pathway. The finding was particularly noteworthy as it suggested that treating SIBO might not only have alleviated gastrointestinal symptoms but also had beneficial effects on related psychiatric conditions. Ning et al. (54) suggested that the urine level of 5-HIAA and lactulose hydrogen breath test result correlation could be used as a potential diagnostic marker for SIBO-related psychiatric manifestations.

Recent research highlights the potential role of tryptophan metabolism dysregulation in the etiology of Parkinson’s disease (PD) (55). Li et al. (55), reviewing 11 studies with 973 participants, found that 46% of patients with PD were SIBO-positive. Patients with PD present a significantly higher, 5.22, odds ratio of SIBO occurrence compared to control patients, suggesting a strong correlation between the two conditions, and the basis of this correlation is a change in the level of neurotransmitter metabolism. Lowering kynurenic acid levels might inadvertently shift the balance of metabolites in the KYN pathway toward the production of neurotoxic metabolites, like quinolinic acid. Kynurenic acid is neuroprotective, and its deficiency can enhance neurotransmission mediated by glutamate, reduce antioxidant capacity, and favor the production of neurotoxic metabolites, potentially leading to neuronal loss (56, 57). Reduction of kynurenic acid levels also decreases the limit of excitotoxicity. Furthermore, reduced kynurenic acid levels can be responsible for 1-methyl-4-phenyl-1, 2, 3, 6-tetrahydropyridine (MPTP) and 6-hydroxydopamine (6-OHDA) toxicity, which may lead to Parkinson’s disease development (58). The altered serotonergic system contributes to the development of motor (slowness of movement, stiffness in the muscles, rhythmic limbs shaking observed at rest, lack of balance and coordination) and non-motor (insomnia, REM sleep disorder, or excessive daytime sleepiness) symptoms from the nervous system and also the symptoms from the digestive system (59). Small intestine bacterial overgrowth was detected in about a quarter of patients with PD, including those recently diagnosed with the disease. Interestingly, SIBO in these patients was not linked to worsened gastrointestinal symptoms, but it was independently associated with poorer motor function. This suggests that SIBO potentially may impact the progression of motor symptoms in PD (60). Additionally, the prevalence of SIBO in patients with PD has been estimated to range between 25–55%, with a higher prevalence in cohorts with more severe PD. This supports the idea that SIBO might be more common in patients with PD, especially as the disease progresses. The study also noted that SIBO can lead to inflammation of the intestinal lining, potentially causing malabsorption of medications like levodopa, a key treatment for PD. This could result in worsened motor function and unpredictable motor fluctuations in patients with PD (61). Another study assessing the prevalence of SIBO in Chinese patients with PD and the potential impact of SIBO on gastrointestinal symptoms and motor function showed that SIBO was highly prevalent in patients with PD as nearly one-third had SIBO, and it was associated with worse gastrointestinal symptoms and worse motor function. In this study, 65 adult patients with PD who exhibited various functional gastrointestinal symptoms of unknown pathology were diagnosed. Thirty-three percent of them were diagnosed with SIBO (62). Similarly, in another group of 39 patients with PD but without type 2 diabetes, a disease affecting the GI tract, or using drugs impacting the intestinal microbiota, a significantly higher proportion, 54.3%, tested positive for SIBO (63). This indicates a notable prevalence of SIBO among patients with PD, even in the absence of other GI diseases. Current research is lacking studies specifically examining all tryptophan metabolites in the serum of patients with SIBO. This gap is critical as tryptophan, kynurenine, and kynurenic acid levels are decreased in the serum of patients with PD. Moreover, in the striatum of patients with PD, decreased kynurenic acid levels are accompanied by an increase in neurotoxic quinolinic acid (64). Also, the TRP/KYN and KA/TRP ratios are significantly higher in the frontal cortex of patients with PD. Additionally, kynurenic acid levels are also reduced in the cortical, caudate, and cerebellum regions of patients with PD (65). The mentioned studies highlight the complex interplay between tryptophan metabolism and PD, and how tryptophan metabolism imbalance may have significant implications for PD progression.

Alzheimer’s disease (AD) is a condition characterized by a progressive disorder of memory and behavior (66). Kowalski et al. (67), studying 45 patients (9 male, 36 female) with dementia in the course of AD, found that 49% of them were SIBO-positive. The inclusion criteria in this study assumed no concomitant central nervous system disorders or GI track diseases and no drug use affecting the results of the hydrogen breath test. Interestingly, only 22% out of 27 patients from the control group were diagnosed with SIBO (67). Within the array of mechanisms implicated in the pathogenesis of Alzheimer’s disease, impaired clearance of amyloid-beta (Aβ) peptides is posited as a critical pathological contributor (68). Neprilysin (NEP), a principal metalloproteinase, orchestrates the catabolism and removal of Aβ peptides within the cerebral milieu. The expression of neprilysin can be affected by products of tryptophan metabolism (69). The hyperphosphorylation of tau protein is mediated by quinolinic acid and leads to neurofibrillary tangles (NFTs) which contribute to neurotoxic protein pathology. Tryptophan metabolites also affect neuroinflammation (70). Beta-amyloid aggregates levels in cerebrospinal fluid (CSF) correlated with kynurenine, kynurenic and quinolinic acid levels (71). Kynurenic acid and quinolinic acid levels were elevated in CSF, but kynurenic acid level was decreased in serum (64). A similar relationship was shown in another study. The meta-analysis by Fernandes et al. (72) examined the KYN pathway in Alzheimer’s disease by analyzing both central and peripheral levels of its metabolites. The study found alterations in the KYN pathway in AD, including decreased tryptophan in peripheral blood, increased kynurenine-to-tryptophan ratio in peripheral blood, decreased 3-hydroxykynurenine in cerebrospinal fluid, and increased kynurenic acid in cerebrospinal fluid, but decreased in peripheral blood. These findings suggest a shift towards the KYN pathway in AD, indicating its potential role in the disease’s pathology (72). A pivotal study (73) demonstrated that 5-hydroxyindoleacetic acid (5-HIAA), the primary metabolite of serotonin, significantly reduces amyloid-beta (Aβ) levels in the brain of a mouse model of AD, as well as in a mouse model with phosphoramidon-induced neprilysin (NEP) inhibition in the brain. Treatment with 5-HIAA not only decreased Aβ accumulation but also improved memory outcomes in APPSWE mice, suggesting that targeting tryptophan metabolism pathways may have a therapeutic potential in AD management. This study underscores the potential of modulating tryptophan metabolism to ameliorate AD symptoms and pathology, offering a novel perspective on the gut-brain axis in neurodegenerative diseases (73).

Tryptophan metabolites are also crucial in processes leading to depression. Major depressive disorder is characterized by an imbalance between neurotoxic quinolinic acid and neuroprotective kynurenic acid. Peripheral activation of indoleamine 2,3-dioxygenase can lead to parallel activation of the KYN pathway in the CNS (74). In depression, serotonin deficiency can be caused by a shunt of TRP towards the KYN pathway (75). Chojnacki et al. (7) assessed patients with SIBO using the Hamilton Depression Rating Scale (HAM-D) and the Hamilton Anxiety Rating Scale (HAM-A) and found that they more often had depression and anxiety than healthy controls (7). Moreover, patients with SIBO more often present character traits such as a negative worldview and suffer more from stress, which may lead to depression (76). On the other hand, gut microbiota disorders in patients with SIBO also may lead to depression and anxiety. Serum levels of tryptophan, kynurenine, kynurenic acid, and also the KA/QA ratio are decreased in patients with depression. Quinolinic acid in CSF was found to be significantly increased in patients with depression attempting suicide (77). Another study also showed a decreased level of the KA/QA ratio and elevated quinolinic acid levels in the serum of depressed patients (78). The plasma KA/QA ratio positively correlated with the hippocampal and amygdalar volume, which indicated that a reduction in this ratio could reduce volumes of these structures (78) and impair the autobiographical memory recall in patients with major depressive disorder (78, 79). Elevated levels of quinolinic acid and changes in serotonin levels can factor in depression development (80, 81). The microbiota dysbiosis causing systemic inflammation can also be linked with depression (82).

Schizophrenia is a mental disorder caused by impaired conversion of tryptophan to serotonin, characterized by symptoms like disruptions in thought processes, emotional responsiveness, or social interactions (83, 84). Erhardt et al. (85) proposed the kynurenic acid hypothesis of schizophrenia, in which increased concentration of kynurenic acid causes alterations in glutamatergic and cholinergic, and indirectly, in dopaminergic signaling, hereby leading to symptoms of schizophrenia (85). Kirkpatrick and Miller stated that inflammation caused by elevated quinolinic acid levels may also play a mechanistic role in schizophrenia pathogenesis (86). However, more studies on the subject are required to fully understand this process. So far, no studies focusing on SIBO and schizophrenia co-occurrence have been performed, but literature indicates a potential link between dysbiosis of the intestinal flora and schizophrenia pathogenesis. Cao et al. meta-analysis about the KYN pathway and potential dynamic changes of kynurenine in schizophrenia showed that the tryptophan level is decreased and the KYN/TRP ratio is increased in patients with schizophrenia (87). Furthermore, lower kynurenine levels are associated with medication-free people suffering from schizophrenia, while higher levels with patients after antipsychotic treatment (87). Elevated kynurenine levels have also been reported in post-mortem brains of patients with schizophrenia (88). Almulla et al. meta-analysis of tryptophan catabolite and kynurenine pathway in schizophrenia showed that kynurenic acid production in the CNS is increased and the neuroprotective products of the KYN pathway outweigh neurotoxic products in patients with schizophrenia (89). Kindler et al. (90) showed that patients with schizophrenia present elevated kynurenic acid levels and the KYN/TRP ratio in the prefrontal cortex. The KYN/TRP ratio in serum can also be elevated, and the changes in tryptophan products can be caused by proinflammatory cytokines (90). Lastly, changes in gut microbiota can also increase risk factors for schizophrenia development (91).

Autism Spectrum Disorder (ASD) is recognized as a complex developmental disability that manifests through challenges in social interaction, communication, and the presence of repetitive or restricted behaviors (92). Recent research suggests that in children with ASD, the behaviors might influence gut microbiome changes rather than the microbiome is influencing ASD. The study analyzed microbiome features, dietary habits, and stool consistency among children with ASD and controls, and found that behavioral aspects of ASD, such as dietary variety, had a stronger association with gut microbiome characteristics than ASD diagnosis itself (93, 94). Moreover, higher levels of certain bacteria, such as *Clostridium* and *Ruminococcus*, correlate with ASD severity (95). Patients with ASD have elevated kynurenine and quinolinic acid levels, but decreased tryptophan levels in the serum (80), which is consistent with the results obtained for patients with SIBO (7, 52). Elevated serotonin levels occurring in patients with SIBO can also increase the risk of autism occurrence (96). Research by Wang et al. (97) on children born in Beijing showed that 96 out of 310 children diagnosed with ASD suffered from SIBO, which translates into a 31% SIBO prevalence rate in children with ASD compared to 9.3% among non-ASD controls. Moreover, SIBO seems to be associated with worse ASD symptoms, such as greater difficulties with social interaction, more pronounced communication challenges, an increase in repetitive behaviors, or heightened sensitivity to sensory inputs (97). Other research, comparing TRP metabolism products between 27 children aged 3-13 and diagnosed with ASD to 15 healthy controls, showed that patients with ASD presented elevated kynurenine production and KYN/TRP ratio, and significantly increased quinolinic acid levels compared to healthy controls (98).

Liu et al. found potential causality from intestinal metabolites in autistic spectrum disorder. They have demonstrated that increased serotonin levels can elevate the risk of autism (99). Another study demonstrated that one in four people on the spectrum has high blood serotonin levels (96). Amulla et al. (100) performed a systematic review and meta-analysis on the tryptophan catabolite or KYN pathway in patients with ASD, evaluating the peripheral levels of tryptophan, its metabolites, and activity in blood and urine samples of patients with ASD. This comprehensive analysis included 25 original research with a total of 6653 participants. The analysis did not find significant differences in blood tryptophan levels or tryptophan-to-competing amino acids ratio between patients with ASD and control patients, nor significant differences in blood kynurenine and kynurenic acid levels. The results of the analysis suggest that abnormalities in peripheral blood tryptophan metabolism or tryptophan catabolite production do not contribute significantly to ASD pathophysiology (100).

## 4 Impact of thyroid function on SIBO: exploring the connection with mental and gastrointestinal disorders

The relationship between hypothyroidism, psychiatric disorders, and gut microbiota is of increasing interest in medical research. Certain gut microbiota can modify thyroid hormone levels by affecting their conversion or degradation (101, 102). This interaction is essential in understanding the pathophysiology of thyroid disorders and gastrointestinal conditions like SIBO (103). Wang et al. (104), utilizing a two-sample Mendelian randomization approach to evaluate the causal relationships between gut microbiota and hypothyroidism, analyzed data from large genome-wide association study meta-analyses. Their findings suggest that specific gut microbiota may be associated with the risk of hypothyroidism, and highlight the impact of gut microbiota on thyroid health (104). Lauritano et al. (30), studying a group of 50 people with hyperthyroidism, found that more than half (54%) suffered from SIBO, while in the control group, consisting of 40 people without hyperthyroidism, only 5% suffered from SIBO (30). Unfortunately, the causal relationship between SIBO and thyroid disorders is not clear yet (26). It is speculated that the SIBO onset in hyperthyroidism may be caused by the thyroid hormones modulating the intestinal motility components: smooth muscles and innervation (105). Chojnacki et al. found that patients with a constipation form of SIBO presented elevated TSH levels and reduced FT3 and FT4 levels in the blood, compared to healthy subjects and patients with the diarrheal form of SIBO (26). Lowered TSH and elevated FT4 and FT3 levels may have a diagnostical value and indicate hypothyroidism (26).

The concentration of antibodies against thyroid peroxidase (ATPO) in the blood is an important indicator of thyroid pathology. It reflects the autoimmunity of the thyroid gland and helps diagnose Hashimoto’s disease (106). ATPO levels are significantly higher in patients with a diarrheal form of SIBO, and patients with a constipation form of SIBO than in healthy patients (26). Knezevic et al. (107) updated systematic review investigated the intricate relationship between gut microbiota and various thyroid diseases, including Graves’ disease, Hashimoto’s thyroiditis, and differentiated thyroid cancer. Their review provides a comprehensive overview of the current understanding of how the gut microbiota influences thyroid function and highlights its significance in the pathogenesis and potential treatment strategies for these thyroid diseases. Researchers have demonstrated that dysbiosis is commonly associated with thyroid disorders, impacting thyroid function through mechanisms, such as inflammation, immune response alteration, and increased intestinal permeability. This imbalance in gut microbiota affects thyroid hormone levels directly through microbial deiodinase activity and influences the absorption of essential minerals like iodine, selenium, zinc, and iron, crucial for thyroid health (108–111). Additionally, the availability of these minerals can further modulate gut microbiota composition and, conversely, be influenced by it, highlighting the intricate relationship between gut health and thyroid function (107).

In the context of Graves’ disease and Hashimoto’s thyroiditis, which are primarily autoimmune, the gut microbiota appear to play a crucial role in modulating an individual’s immune responses (112, 113). This is particularly relevant given the established connection between gut health and immune function (114). The composition and diversity of the gut microbiota can influence systemic inflammation and immune tolerance, factors that are critical in the development and progression of autoimmune thyroid diseases, leading to Graves’s disease (115). Research indicates that the presence of *Bacillus*, *Blautia*, and *Ornithinimicrobium* could serve as potential biomarkers for differentiating between Graves’ disease and Hashimoto’s thyroiditis from healthy individuals. Additionally, these findings suggest a possible role for the “ABC transporter”(responsible for ATP transport) metabolic pathway in the etiology of Graves’ disease and Hashimoto’s thyroiditis (115). McGaffe et al. (116) emphasized that hypothyroidism can often be misdiagnosed as a psychiatric illness. Patients with hypothyroidism may present with depression, organic mental disorder, apathy, or even frank psychosis (116). The research underlined the importance of thyroid function screening in patients presenting with psychiatric symptoms, as psychiatric manifestations related to hypothyroidism can improve with thyroid hormone replacement therapy unless the disease progresses enough to cause irreversible brain damage (116). Historically, alterations in thyroid function have been linked to mood disturbances, with hypothyroidism often associated with depressive states and hyperthyroidism linked to manic behaviors (117).

Studies show a strong association between SIBO and depression (118, 119). Changes in the gut microbiome can lead to depressive disorders due to hormonal changes, among other things (76, 120). Such hormones include thyroid hormones, which can cause several psychiatric disorders as a result of thyroid disease (121, 122). It has been shown that hypothyroidism or autoimmune thyroid diseases, such as Hashimoto’s disease, can promote symptoms of depression, anxiety, or bipolar affective disorder (27, 28, 122, 123). Patients with Hashimoto’s have been shown to have a sixfold increase in risk of depressive symptoms (29), a fact that confirms that there is a strong association between elevated ATPO and depression (124).

Hypothyroidism can contribute to sexual dysfunction in men (59-63% of hypothyroid patients) and women (22-46% of hypothyroid patients). In men, these disorders may manifest as delayed ejaculation, while in women they may manifest as decreased libido (125). Hypothyroidism may also be related to sleep disorders (22). People with reduced levels of thyroid hormones have longer sleep latency, shorter sleep, and less satisfaction with sleep quality (126). Additionally, symptoms that occur with hypothyroidism or autoimmune diseases, such as constipation, abdominal pain, paresthesias, decreased tolerance to low temperatures, and muscle cramps (127) may contribute to insomnia (22). An underactive thyroid can lead to slow gut motility, which may contribute to SIBO development (31). This, in turn, can lead to gastrointestinal discomfort, potentially exacerbating sleep disturbances like insomnia (128, 129). The interconnectedness of these conditions highlights the complexity of the gut-thyroid-sleep axis, but no scientific publication indicated SIBO as a direct cause of insomnia.

These studies collectively suggest a significant interplay between gut microbiota, thyroid function, and mental health. They underscore the potential for gut microbiota as a therapeutic target in hypothyroidism and related psychiatric disorders. Modulating the gut microbiota through diet, probiotics, and other means could offer new options for treating and managing these conditions (107, 130). Furthermore, understanding the gut-thyroid axis may provide insights into individual susceptibilities to thyroid disorders and pave the way for personalized medicine approaches.

## 5 Discussion and conclusions

**Figure 2:**
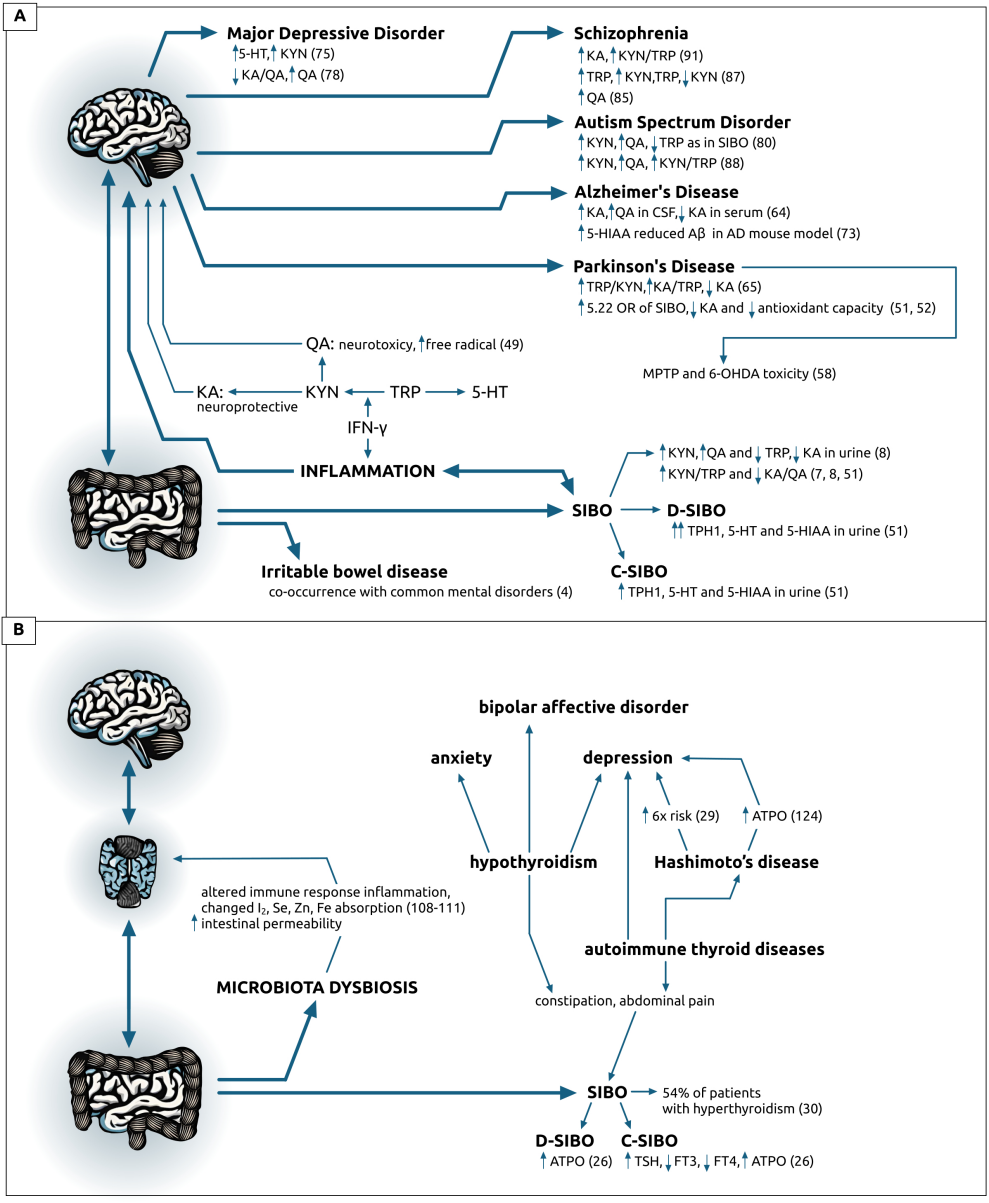
Visual representation of data on: **(A)** brain-gut axis described as the altered tryptophan metabolism in patients with diagnosed small intestine bacterial overgrowth (SIBO) or the irritable bowel syndrome (IBL) and various neurodegenerative and psychiatric disorders, **(B)** on brain-thyroid-gut changes causing or co-occurring with microbiota dysbiosis or thyroid pathologies and common psychiatric disorders. Data on **(B)** origin form references 27-29 and 101-130, unless specifically indicated. 5-HIAA, 5-hydroxyindoleacetic acid; 5-HT, serotonin; 6-OHDA, 6-hydroxydopamine; AD, Alzheimer’s Disease; ASD, Autism Spectrum Disorder; ATPO, antibodies against thyroid peroxidase; Aβ, amyloid-beta; CSF, cerebrospinal fluid; C-SIBO, constipation SIBO; D-SIBO, diarrheal SIBO; FT3, free triiodothyronine; FT4, thyroxine; IBL, irritable bowel disease; IFN-γ, interferon gamma; KA, kynurenic acid; KYN, kynurenine; MDD, Major Depressive Disorder; MPTP, 1-methyl-4-phenyl-1; 2; 3; 6-tetrahydropyridine; PD, Parkinson’s Disease; QA, quinolinic acid; SIBO, small intestinal bacterial overgrowth; TPH, tryptophan hydroxylase; TRP, tryptophan; TSH, thyroid stimulating hormone.

The presented mini-review tries to link the relationship between the small intestinal bacterial overgrowth (SIBO), gut bacteria, and clinical outcomes, assess and synthesize all available literature on the interaction among SIBO, tryptophan levels, thyroid hormonal levels, and the KYN signaling pathway relative to neurologic or psychiatric disorders (**Figure 2**). New research could explain how SIBO impacts neurologic and mental disorders and could potentially lead to new therapeutic strategies that target both gut-brain systems and gut microbiome for improved clinical management and better treatment outcomes.

Reviewed studies indicate a significant link between altered tryptophan metabolism in SIBO patients and the development of various neurodegenerative and psychiatric disorders. The dysregulation in tryptophan pathways, particularly the imbalance between neurotoxic and neuroprotective metabolites, is implicated in the pathology of disorders such as Parkinson’s and Alzheimer’s disease, depression, schizophrenia, and autism spectrum disorder. These findings highlight the importance of tryptophan metabolism in the gut-brain axis and suggest that addressing SIBO and its impact on tryptophan metabolism could be a potential therapeutic avenue for managing and understanding these complex disorders.

It’s reasonable to hypothesize that focusing on correcting gut dysbiosis rather than solely relying on pharmacotherapy might be advantageous in treating neurodegenerative and psychiatric disorders. This perspective stems from the increasing evidence supporting the critical role of the gut-brain axis in these conditions. The disturbances in neurotransmission, as observed in the altered tryptophan metabolism in SIBO and other gut-related disorders, underline the potential impact of gut health on neurological and psychiatric well-being. Consequently, therapeutic strategies that aim to restore healthy gut microbiota could prove to be a pivotal component that effectively manages these complex disorders, potentially offering a more holistic and root-cause approach to treatment.

Despite the increasing interest in researching the connection between small intestinal bacterial overgrowth (SIBO) and psychiatric disorders and despite increasing evidence supporting the critical role of the gut-brain axis in these conditions significant gaps in the knowledge still exist. Firstly, the precise mechanisms underlying the interaction between SIBO and the development or exacerbation of psychiatric conditions are not fully understood. Particularly, those associated with the tryptophan pathway, such as autism, schizophrenia, Alzheimer’s, and Parkinson’s diseases. There is a need for more detailed studies that specifically investigate the influence of SIBO on the metabolism of tryptophan and its downstream effects on neurotransmitter production and brain function. Additionally, while the correlation between thyroid disorders and psychiatric illnesses is recognized, the role of SIBO in this relationship is less clear. There is a lack of research exploring how SIBO might affect thyroid function and, in turn, how this interaction could influence psychiatric disease processes. This gap is particularly critical considering the potential implications for treatment strategies that target not just the psychiatric symptoms, but also the underlying gut and thyroid dysfunctions. Moreover, there is a scarcity of longitudinal studies that could provide insights into the temporal relationships between SIBO, gut dysbiosis, and the onset or progression of psychiatric disorders. Such studies are essential to determine whether SIBO is a cause, consequence, or coincidental occurrence relative to these mental health conditions.
